# Editorial: Embodiment and Co-adaptation Through Human-Machine Interfaces: At the Border of Robotics, Neuroscience and Psychology

**DOI:** 10.3389/fnbot.2022.871785

**Published:** 2022-03-23

**Authors:** Philipp Beckerle, Claudio Castellini, Bigna Lenggenhager, Strahinja Dosen

**Affiliations:** ^1^Department of Electrical Engineering, Friedrich-Alexander-Universität Erlangen-Nürnberg, Erlangen, Germany; ^2^Department of Artificial Intelligence in Biomedical Engineering, Friedrich-Alexander-Universität Erlangen-Nürnberg, Erlangen, Germany; ^3^DLR–German Aerospace Center, Institute of Robotics and Mechatronics, Weßling, Germany; ^4^Department of Psychology, University of Zurich, Zurich, Switzerland; ^5^Department of Health Science and Technology, Aalborg University, Aalborg, Denmark

**Keywords:** human-machine interfaces, sensorimotor control and learning, brain plasticity, constructivist psychology, cognitive models, embodiment, agency

## Introduction

Traditionally, robotics and psychology have little to share; at least, if we think of robotics as an endeavor to build machines able to *autonomously* perform tasks that are undesirable or impossible for human beings. Nevertheless, besides addressing safety requirements for close physical interaction, which are tackled by approaches like soft robotics and impedance/admittance control (Albu-Schäffer et al., 2008; Kim et al., 2013; Schumacher et al., 2019), the two fields gradually approached each other over the recent years. Together with the surge of virtual and extended reality technologies that can provide immersive environments, this opens up an exceptional opportunity to the scientific community: that of studying the human being via *human-robot interaction*, for which joint competencies from robotics, neuroscience, and psychology are critical (Rognini and Blanke, 2016; Beckerle et al., 2018a).

Driven by two specific concepts encircled by the aforementioned idea, namely embodiment and co-adaptation, we have launched this Research Topic (RT). In this context, we understand embodiment as something being “experienced as a part of the body schema due to multisensory integration” (Nostadt et al., 2020) and co-adaptation as the robot learning to align to “its human operator/user while the human adapts to it” (Beckerle et al., 2018a). Especially in the fields of rehabilitation and assistive robotics, where the robotic device is physically attached to the user's body, the tighter integration between user's needs and system design is particularly critical. To what extent a robot can, should or should not feel like a part of the user's bodily self through mutual adaptation has been increasingly investigated and discussed (Makin et al., 2017; Beckerle et al., 2018a). This human-centered approach appears crucial for assistive robotics and requires methodological instruments and knowledge from human psychology, e.g., theories of constructivist psychology, and insights into multisensory foundations of the bodily self and human motor control (Makin et al., 2017; Beckerle et al., 2018b; Niedernhuber et al., 2018).

We are happy to present and discuss the essence of the 14 contributions that we collected in this RT, which all concentrate on embodiment, co-adaptation, and bidirectional human-machine interfaces. Through joint contributions from engineers, neuroscientists, and psychologists, this RT shows a remarkable level of interdisciplinarity, which we deem necessary to tackle this research area. We hope that this RT will boost such research and provoke thoughts and ideas in young and established scientists alike, with the aim of building further bridges between different disciplines.

## Embodiment

A central question for assistive robotics is to what extent the robot is integrated into the user's body schema and body image. However, to answer this question, we first need to understand the integration processes and elucidate factors that can affect the feeling of embodiment as well as develop methods to modulate it. In a perspective article, Matamala-Gomez et al. present ideas for a new rehabilitation approach that employs full virtual body-ownership illusions, using 360° videos to assess and modulate the representation of the impaired limb, to improve motor rehabilitation of stroke patients. They put forward that such “positive technology” could precede more conventional motor rehabilitation methods and normalize a distorted body schema and image. Barresi et al. assess whether modulating the psychophysiological state through controlled breathing affects the feeling of embodiment induced by an experimental protocol akin to the virtual hand illusion. Their results indicate that slowing down breathing pace using online biofeedback of respiratory rate seems to induce stronger embodiment of the virtual hand compared to the condition with normal breathing rate. Their study emphasizes that embodiment is indeed a complex experience that depends on multiple factors, including interoceptive processes. Bekrater-Bodmann also investigates the multifaceted nature of this phenomenon by elucidating factors associated with the embodiment of a lower limb prosthesis. His findings point to the particular importance of subjective-evaluative variables related to how a person perceives the amputation and the device, also revealing a positive relationship between embodiment and user satisfaction. Similarly, Sturma et al., who investigate the body image pre and post elective amputation and prosthetic reconstruction in a longitudinal study in patients with brachial plexus injuries, stress the huge interindividual differences in the patient's sense of embodiment. Nevertheless, their data suggests a more positive body image 2.5 years after the surgical procedure. Middleton and Ortiz-Catalan use deep semi-structured interviews with three prosthesis users to elucidate personal and social implications of living with an upper limb prosthesis. From a medical anthropology perspective, the study shows that the relation between the users and their bionic limb is subject-dependent, complex, and constantly evolving. They find a tight coupling between the quality of prosthesis use in daily life and users' self-esteem, self-image, and incorporation of the device into the body.

## Co-Adaptation

Embodiment is likely shaped by a process of adaptation and tight interaction between the user and the machine. If the system can also adapt to the user, e.g., as in prosthetics (Hahne et al., 2017), this process is called mutual adaptation or *co-adaptation*. Some of our authors have hereby tried to define and determine, measure, and quantify co-adaptation, in order to draw a path toward fostering and exploiting it. Studying co-adaptation in a team, van Zoelen et al. engage 18 participants in a cooperative human-robot task and define co-adaptation as a rather fast, changing attitude in human-robot interaction. This model enables them to recognize four categories of interaction (stable, sudden, gradual, and active), which are denoted as *interaction patterns*. De Santis takes a more quantitative approach and proposes a general framework for user-machine interaction. The problem is explicitly formulated as a closed-loop block diagram, monitoring the change in the parameters of both the user and a machine to define co-adaptation. Schofield et al.'s perspective argues that embodiment in myoelectrically-controlled prostheses is the key to achieve optimal control and user satisfaction. Tool incorporation, agency and ownership are the three pathways the authors identify to achieve, in the long run, an embodied bionic limb.

## Human-Machine Interfaces

To facilitate embodiment and co-adaptation during human-robot interaction, the implementation of suitable interfaces between human and machine is a challenge of crucial relevance (Beckerle et al., 2018a). For efficient communication between the interacting partners, these interfaces must inevitably be *bidirectional*. They need to enable the user to send commands to the system but they also need to convey sensor data from the device back to the user, thereby closing the control loop. Accordingly, Moore et al. investigate the question of how to convert haptic feedback from prosthetic fingertips into vibrotactile feedback provided to another part of the participants' bodies. They conclude that embodiment was similar for natural feedback compared to providing proximal vibrotactile feedback. Cansev et al. review neurophysiological and psychological design criteria to create haptic interfaces that can mediate affective touch and derive recommendations for interface design. To enable this, future bidirectional human-machine interfaces need to transmit slow and low-force motion or force/torque patterns and consider their relation to the users' experiences. Mouchoux et al. investigate how different schemes for the integration of volitional and automatic control influence the performance and usability of a semi-autonomous prosthesis. The study finds that all semi-autonomous schemes increased the performance with respect to the purely manual control. However, the study also reveals that the specific approach to integrating automatic and manual control is an important factor for the design of a semi-autonomous prosthesis, as different schemes resulted in different performance, especially when automatic control was less reliable. Beyond this, Falandays et al. examine joint decision-making in human-machine interfaces and how choices are influenced by the characteristics of the provided response options. Their results imply that users will often begin acting before their cognitive choice has been finalized, and in addition, synergies between humans and machines are reported.

## Interdisciplinary Perspectives

Wudarczyk et al. contributed an exquisitely meta-scientific paper relating to the lessons learned during an interdisciplinary project. We cannot but agree with most of their claims, such as, e.g., the necessity of finding common goals, agreeing on publication outlets and a common language, reciprocally transferring technology and discussing the differences in research practices belonging to different fields. Lastly, in a short but dense contribution, Bettoni et al. propose that radical constructivism might be used as a unifying framework to design the machine-learning core of a myocontrol system for prosthetics. Elements of this psychological discipline seem particularly suited to the authors to shape the interaction protocols, interface, and channels of myocontrol, with the aim of fostering co-adaptation.

In conclusion, we believe that an interdisciplinary perspective is crucially required to achieve human-machine interfaces that promote embodiment and co-adaptation. Despite such collaborations demand for continuous adjustment between project partners from different domains, the contributions to this RT underline that such interaction is well worth the efforts. Crossing the field boundaries is enriching for all the sides, yields promising results, and is therefore the approach that shall be welcomed and further developed in the next years.

## Author Contributions

All authors listed have made a substantial, direct, and intellectual contribution to the work and approved it for publication.

## Funding

This work was supported by the DFG (German Research Agency) projects Deep-Hand (grant no.: CA 1389/1-2) and TRAIN (grant no.: BE 5729/16-1), project ROBIN (grant no: 8022-00243A) funded by Independent Research Fund Denmark as well as the Swiss National Science Foundation (grant no.: PP00P1_170511).

## Conflict of Interest

The authors declare that the research was conducted in the absence of any commercial or financial relationships that could be construed as a potential conflict of interest.

## Publisher's Note

All claims expressed in this article are solely those of the authors and do not necessarily represent those of their affiliated organizations, or those of the publisher, the editors and the reviewers. Any product that may be evaluated in this article, or claim that may be made by its manufacturer, is not guaranteed or endorsed by the publisher.
